# Diagnosis and Treatment of Rheumatic Adverse Events Related to Immune Checkpoint Inhibitors

**DOI:** 10.1155/2020/2640273

**Published:** 2020-08-04

**Authors:** Yan Xiao, Lin Zeng, Qinglin Shen, Zhiyong Zhou, Zhifang Mao, Qin Wang, Xiquan Zhang, Yingliang Li, Weirong Yao

**Affiliations:** ^1^Department of Oncology, Jiangxi Provincial People's Hospital Affiliated to Nanchang University, Jiangxi, China; ^2^Department of General Surgery, The First Affiliated Hospital of Nanchang University, Jiangxi, China

## Abstract

Immune checkpoint inhibitors (ICIs) have completely changed the treatment of cancer, and they also can cause multiple organ immune-related adverse reactions (irAEs). Among them, rheumatic irAE is less common, mainly including inflammatory arthritis, rheumatic myalgia/giant cell arteritis, inflammatory myopathy, and Sjogren's syndrome. For oncologists, rheumatism is a relatively new field, and early diagnosis and treatment is very important, and we need to work closely with experienced rheumatologists. In this review, we focused on the incidence, clinical characteristics, and treatment strategies of rheumatic irAE.

## 1. Introduction

In recent years, immune checkpoint inhibitors (ICIs) have made significant breakthroughs in cancer treatment. A large number of clinical trials at home and abroad have confirmed that ICIs is a broad-spectrum, long-lasting, safe, and effective antitumor drug [1]. It can inhibit and kill tumor cells by enhancing the antitumor immune function of the body. It has shown a remarkable clinical effect in the treatment of many kinds of malignant tumors. At present, it has been approved by FDA for melanoma, lung cancer, renal cell carcinoma, Hodgkin's lymphoma, head and neck tumor, and urothelial carcinoma [2]. According to its mechanism of action, ICIs can be divided into three categories: programmed cell death protocol 1 (PD-1), programmed cell death protocol ligand 1 (PD-L1) inhibitors, and cytotoxic T-lymphocyte-associated antigen-4 (CTLA-4) inhibitors [3, 4].

ICIs can enhance the antitumor effect of T cells by blocking the negative regulatory signals of T cells and also affect the immune tolerance of human normal tissues, resulting in immune-related adverse events (irAEs) [5, 6]. irAEs are very common in clinic, and they can occur in almost any organ during or after the treatment of ICIs, generally involving the skin, digestive tract, and endocrine system, but rheumatic irAEs seem to be less common [7–9]. In clinical practice, the common terminology criteria adverse events (CTCAE) are usually used to grade irAEs [10, 11] (for details, see Table 1). The severity is divided into G1: mild or asymptomatic; no intervention is needed; G2: moderate, affecting instrumental activities of daily living (ADL), such as shopping; limited intervention is needed; G3: serious, medical events, limiting self-care ADL, requiring hospitalization; G4: life-threatening events, requiring emergency treatment; and G5: deaths related to adverse reactions. Although most of the irAEs are levels 1 to 2, there is still 0.5%-18.0% in more than level 3 of adverse reactions, even life-threatening [12].

Rheumatic irAEs are different from other organs' irAEs and traditional autoimmune diseases. Rheumatic irAEs can persist for a long time even after termination of treatment [13, 14]. Rheumatic irAEs have a wide range of manifestations, mainly including inflammatory arthritis (IA), polymyalgia rheumatica (PMR)/giant cell arteritis (GCA), inflammatory myopathy (IM), and Sjogren's syndrome (SS). These irAEs are mainly described in patients without autoimmune diseases in the past. However, it has also been reported recently that patients with underlying rheumatic diseases have relapsed and developed new irAEs after receiving ICI treatment.

At present, clinicians have a relatively poor understanding of rheumatic irAEs. Although there are few fatal complications, it will significantly affect the functional activities of cancer patients and limit the use of ICIs [13]. Therefore, this review will focus on the pathogenesis, incidence, clinical characteristics, and treatment strategies of rheumatic irAEs.

## 2. Pathogenesis of Rheumatic irAEs

CTLA-4 and PD-1/PD-L1 are important immunosuppressive molecules in the immune system, which can inhibit the activation of effector T cells and maintain the balance between the activation and inhibition of T cells. CTLA-4 acts on the early stage of T cell activation, while PD-1/PD-L1 inhibits the activated T cells in the effective stage [15]. ICIs can enhance the antitumor activity by blocking CTLA-4 and PD-1/PD-L1 to activate effector T cells, especially CD8^+^ T cells, and destroying the signal transduction pathway that inhibits tumor immunity [16]. At present, there are few studies on the pathogenesis of irAEs. The possible mechanisms can be summarized as follows: (1) overactivation of effector T cells, (2) reduction of regulatory T cell function, (3) large-scale release of IFN-*γ* and TNF, (4) toxic effects of macrophages and neutrophils, and (5) antibody produced by B cells. Therefore, further research is needed to explore the potential mechanism of rheumatic irAEs.

## 3. Incidence of Rheumatic irAEs

The mechanism of action of different types of ICIs is different, which leads to different incidence of irAEs. In general, the incidence of irAEs of CTLA-4 mAb was higher than that of PD-1/PD-L1 mAb [17]. A meta-analysis showed that the prevalence of irAEs with CTLA-4 monoclonal antibody could be as high as 75% [18], while the prevalence of irAEs with PD-1 and/or PD-L1 monoclonal antibody was about 30% [19]. In addition, CTLA-4 mAb has more serious toxicity than PD-1/PD-L1 mAb. It has been found that 43% of the patients treated with ipilimumab have level 3 or more toxic events, while less than 20% of the patients are treated with PD-1/PD-L1 mAb [20].

At present, the description of rheumatic irAEs mainly comes from case reports, which is easy to be ignored in clinical practice [21]. So far, the most common symptoms of rheumatic irAEs are arthralgia/arthritis and myalgia/myositis, with the prevalence of 1-43% and 2-20%, respectively [22]. In a phase III clinical trial for melanoma, the incidence of joint pain secondary to ipilimumab was about 5%, the incidence of joint pain secondary to pembrolizumab was 9%-20%, the incidence of joint pain secondary to nivolumab was 5%-16% [23], and the incidence of joint pain treated by ipilimumab and nivolumab combination was 10.5% [24]. According to reports, the incidence of myositis caused by ICIs is 0.15-1.28%, and the probability of concomitant myocarditis is as high as 32.0% [25]. However, in the relevant literature of ICI clinical trials, few studies describe PMR and GCA, and one retrospective study reported that the incidence of PMR caused by ICI treatment was 0.2-2.0% [26, 27]. A case of giant cell arteritis was reported in a phase I clinical trial of ipilimumab ± bevacizumab in the treatment of metastatic melanoma. Dry syndrome caused by ICIs has also been reported recently [28]. In the clinical trial of nivolumab in the treatment of metastatic melanoma, 24% of the patients had dry mouth. In the clinical trial of nivolumab in the treatment of renal cancer, 3.0-11.0% of patients also have dry mouth [29].

In fact, the authenticity of the incidence of rheumatic irAEs is usually limited [13, 14]. On the one hand, the codes of rheumatism/musculoskeletal adverse events used in clinical trials are inconsistent. For example, arthritis can be encoded as joint pain, joint effusion, and musculoskeletal pain. If the AE code is not strictly standardized, the coding of the same symptom may be different. Therefore, the similar encoding can be integrated to better reflect the real incidence rate of rheumatic irAEs. On the other hand, the reason is the classification of CTCAE used in current clinical trials. In many clinical trials, only level 3 adverse events are reported, while rheumatic irAEs are usually mild, so they are easy to be missed. CTCAE classifies many rheumatic adverse events requiring corticosteroid or ICI treatment into level 1 or level 2. The rCTCAE compiled by OMERACT evaluates the applicability of CTCAE used in the field of oncology in the field of rheumatology, reclassifies the coded adverse events, and classifies similar symptoms into higher categories [30, 31]. Therefore, the incidence of rheumatic/musculoskeletal irAEs may be higher if this rCTCAE is used.

## 4. Clinical Characteristics of Rheumatic irAEs

### 4.1. Inflammatory Arthritis (IA)

IA is a group of diseases characterized by arthritis [31]. The clinical manifestations of inflammatory arthritis caused by ICIs are various, which can be divided into two categories: one is similar to rheumatoid arthritis (RA), which mainly affects small joints (metacarpophalangeal joint, proximal interphalangeal joint, and the wrist and knee), which is different from the traditional RA. This kind of patients is not common in women. In the early stage of the disease, tendon involvement is more prominent, and rheumatoid factor (RF) and anti-citrulline peptide antibody (ACPA) are usually negative [32]. Another is similar to spinal arthritis (SPA), which is characterized by oligoarthritis, mainly involving in large joints, such as inflammatory back pain, adhesion point inflammation, and phalangitis [14, 31, 33, 34]. A few patients also have reactive arthritis (conjunctivitis with oligoarthritis, asymptomatic urethritis) and psoriatic arthritis [26, 35, 36]. However, HLA-B27 was not positive in these patients [20].

A number of research results show that the time of joint pain after the application of ICIs is from 2 to 24 months, and the median time of joint pain disappearance is 9.2 ± 6.1 months, but musculoskeletal symptoms may last for more than one year [37–41].

### 4.2. PMR (Polymyalgia Rheumatica)/GCA (Giant Cell Arteritis)

PMR is an inflammatory disease, mostly in the elderly, which mainly occurs in the age of more than 50 years. Its typical clinical features are persistent pain and morning stiffness in the neck, scapula, and pelvic belt, sometimes with systemic symptoms, such as mild peripheral arthritis and dorsal edema [23, 29, 41–43]. The auxiliary examination showed that the inflammatory index was significantly increased, RF and ACPA were generally negative, and low dose hormone (prednisone < 15 mg/D) was effective. The imaging manifestations of bursitis of deltoid muscle and tenosynovitis of the biceps under the shoulder may be the characteristics of RA or PMR [44, 45]. GCA is a kind of vasculitis, which mainly invades the major arteries, such as one or more branches of the carotid artery (especially the temporal artery), accompanied by granuloma formation, which is relatively rare in China [20]. PMR and GCA represent different clinical manifestations in the same disease process. Patients with GCA often have PMR, so the two diseases are often discussed together [46].

After the use of ICIs, the clinical and imaging manifestations of PMR are almost the same as those of traditional PMR. In most cases, RF and ACPA are negative [23, 39, 47, 48]. It can be seen that there is a significant increase in the inflammatory index, but some studies have found that there is no increase in CRP in this part of patients with typical clinical characteristics of PMR [26]. And some patients did not respond to low-dose prednisone [13]. Only a few patients will cause GCA after using ICIs, mainly manifested as headache, tenderness of temporal artery, intermittent lameness of mandible, and transient diplopia. The pathological biopsy findings are consistent with the traditional GCA [47]. It has been reported that the attack time of PMR/GCA secondary to ICIs is 10 days to 1 year [23, 26, 47].

### 4.3. Inflammatory Myopathy (IM)

IM is a group of heterogeneous diseases; the main pathological features are inflammatory cell infiltration and myofibrillar necrosis of skeletal muscle [49]. Different types of IM can involve different target organs such as the skin and muscle, so the clinical manifestations are complex. Among them, idiopathic inflammatory myopathies (IIMS), including polymyositis (PM), dermatomyositis (DM), and inclusion body myositis (IBM), have unknown etiology and are related to autoimmunity. The IM caused by ICIs is relatively rare, and its clinical manifestations are consistent with PM. It is often manifested as myalgia, weakness of proximal muscle, ptosis of the upper eyelid, and elevation of muscle enzyme. Compared with IIMS, the symptoms of such patients are usually sudden attack, usually within two months of ICI treatment, and the symptoms generally last for 5 days to 115 weeks [25, 50].

Different from the traditional IM, the classic dermatomyositis rash caused by ICIs is rare in IM patients. The response of intravenous immunoglobulin may be poor. The specific antibody of serum myositis is usually negative, and the axial part or facial muscles may be involved [51–54]. And the frequency of overlap with myocarditis and myasthenia gravis in these patients was higher than that of traditional IM [55]. Therefore, the diagnosis should be differentiated from tumor-related myocarditis and myasthenia gravis. A small number of patients will also have myositis syndrome with fasciitis [56]. Up to now, there is no case of IM caused by ICIs with the unique histological characteristics of inclusion body myositis. Although the overall incidence of myositis is not high, the mortality rate can reach 17.0%, second only to myocarditis [55]. At the same time, the mortality rate of myocarditis or other neuromuscular diseases is higher, which is usually caused by heart failure or respiratory failure [50, 57]. It is worth noting that there may be cases with normal muscle enzymes or muscle involvement, but no myasthenia or myalgia [58–63]. In view of the high mortality rate of myositis, we should pay attention to the dynamic monitoring of muscle enzyme changes in patients with clinical high suspicion of IM and carry out muscle biopsy if necessary.

### 4.4. Sjogren's Syndrome (SS)

SS is a chronic inflammatory autoimmune disease involving mainly exocrine glands such as salivary glands and lacrimal glands [28]. The main clinical manifestations are dry mouth, dry eyes, rampant caries, and mumps. The serum autoantibody is anti-SSA or anti-SSB (+), and the dry mouth is responsive to sialidase treatment. Warner [64] et al. discussed the clinicopathological characteristics of SS related to ICI treatment, evaluated 20 patients with xerostomia, and found that the median interval between ICI treatment and xerostomia attack was 70 days, mainly manifested as more serious xerostomia symptoms at night or after exercise, hoarseness of voice, change of taste, sensitivity to spicy or acid food, and abnormal parotid swelling or tenderness. Only 2 of them were positive for RA and anti-SSA. Labial salivary gland biopsy (LSGB) showed mild to severe sialitis, which was different from traditional SS, with diffuse lymphocytic infiltration and acinar injury. There were T lymphocyte aggregation in 8 patients, mainly CD3^+^ T cells; PD-1/PD-L1 was positive. In addition, it has been reported that 4 cases of severe salivary hypofunction after treatment with nivolumab, ipilimumab, or combination therapy have been described. Anti-Ro antibody is negative, only 1 case has parotitis, parotid ultrasound shows hypoechoic focus, and anti-LA/SSB antibody indicates positive [28].

## 5. Treatment of Rheumatic irAEs

For the whole process management of rheumatic irAEs, the European Society for Medical Oncology (EMSO), the National Comprehensive Cancer Network (NCCN), and the Chinese Society of Clinical Oncology (CSCO) have developed immune-related toxicity management guidelines according to the consensus of experts. These guidelines were issued by consensus of experts from various disciplines, and no prospective test was conducted [65, 66]. Recent studies have shown that patients with rheumatic irAEs have better prognosis in tumor treatment than other irAEs [26]. Therefore, it is very important for oncologists to properly manage these rheumatic irAEs.

### 5.1. Treatment Strategy of Inflammatory Arthritis

IA is the most common clinical manifestation in rheumatic irAEs. In consideration of this diagnosis, it is necessary to evaluate the joint function and improve the laboratory tests such as ESR/CRP, ANA, RF, and ACPA, and it is feasible to determine the number of joint involvements by joint X-ray, MRI, or musculoskeletal ultrasound. ESMO, NCCN, and CSCO guidelines all provide specific recommendations for the treatment of IA, and the overall treatment principles are basically the same (Table 2). For patients above the G2 level, patients whose symptoms last more than 6 weeks or need more than 20 mg of prednisone per day and cannot decrease to less than 10 mg/D within 4 weeks should be considered to be transferred to rheumatology department [31].

### 5.2. Treatment Strategy of RA and GCA

The incidence rate of PMR and GCA is low. For patients with suspected PMR, ESR and CRP are necessary. Ultrasound examination of the shoulder and buttocks is also necessary. If visual symptoms or headache occurs, then temporal artery ultrasound and biopsy are considered. At present, only suggestions for PMR/GCA treatment are put forward in NCCN guidelines. The treatment principle of PMR is shown in Table 3. The difference between the treatment strategy of GCA and PMR lies in stopping immunotherapy immediately after diagnosis, giving 1 mg/kg/D prednisone and decreasing in quantity within 8-12 weeks. If accompanied by visual symptoms, consider steroid pulse therapy (methylprednisolone 500-1000 mg) and consult with the eye. For GCA, the rheumatology department should be consulted even for mild cases to prevent permanent organ damage [31].

### 5.3. Treatment Strategy of Myositis or Myalgia

IM is also one of the common clinical manifestations of rheumatic irAEs. Once it occurs, the mortality is high. During the diagnosis, we should perfect the examination of creatine kinase/aldolase, troponin, myositis antibody, muscle MRI, and electromyogram; take muscle biopsy if necessary; and focus on the possibility of myasthenia gravis or myocarditis. The ESMO guidelines do not mention the treatment of ICI-related myositis. See Table 4 for the treatment principles of NCCN and CSCO compass for the disease. The treatment principles of the two are slightly different. For G1 patients, NCCN guidelines consider the need to stop immunotherapy, while CSCO guidelines consider whether to continue to use ICIs through a comprehensive assessment of patients' muscle strength. In the actual clinical work, for all suspected cases of myositis, myasthenia, and creatine kinase elevation, we should keep a high vigilance and timely refer to the rheumatology department or neurology department to avoid life-threatening situations [31].

### 5.4. Treatment Strategy of Sjogren's Syndrome and Other Connective Tissue Diseases

There are no specific and detailed treatment suggestions for SS and other connective tissue diseases. The treatment of these diseases mainly refers to the treatment principle of traditional rheumatism and adjusts the dosage of glucocorticoid and immune checkpoint inhibitors according to the severity of organ involvement. Most of the patients with SS described in the case reports were treated with systemic glucocorticoids, with an average dose of prednisone of 40 mg/D, and a few patients with severe disease were treated with IVIG and other immunosuppressive therapy [41, 67, 68]. Pilocarpine, as a muscarinic receptor agonist, can also effectively improve the symptoms of xerostomia. Similarly, other connective tissue diseases can be treated with systemic hormone. For example, in one patient with lupus nephritis after treatment with ipilimumab, renal function was significantly improved after treatment with 1 mg/kg prednisone [69].

## 6. Summary

With the wide application of ICIs, the emergence of irAEs in clinical work has brought many challenges to oncologists but also opened up new research areas. Among them, rheumatic irAEs are relatively rare, mainly including inflammatory arthritis, rheumatic myalgia/giant cell arteritis, inflammatory myopathy, and Sjogren's syndrome. These diseases should be distinguished from traditional rheumatic diseases. At present, irAEs are generally classified by CTCAE and managed according to the standard international guidelines. The therapeutic drugs mainly include NSAIDs, glucocorticoids, and DMARDs. For oncologists, rheumatism is a relatively new field, we must improve the understanding of rheumatic irAEs, understand its pathogenesis, clinical characteristics, and treatment methods.

Although the overall incidence of rheumatic irAEs is not high, we still need to be vigilant. Oncologists need to actively cooperate with experienced rheumatologists to improve the diagnosis rate of rheumatic irAEs, timely referral so as not to delay the treatment opportunity, and at the same time, weigh the advantages and disadvantages to decide whether to continue the treatment of ICIs.

## Figures and Tables

**Table 1:** General terminology standard for adverse event (CTCAE) version 5.0.

Level	Clinical description
1	Mild: asymptomatic or mild; only clinical or diagnostic; no treatment required
2	Moderate: requires minor, local, or noninvasive treatment; age equivalent instrumental ADL limitation
3	Serious or medically significant but not immediately life-threatening Causes hospitalization or prolongation of hospitalization; disability; self-care ADL limitation
4	Life threatening; in need of urgent treatment
5	Death-related AE

**Table 2:** Management of immunology-related informational arthritis.

Level	Description	NCCN/CSCO guideline	ESMO guideline
G1	Mild pain with inflammatory symptoms (improved by exercise or heating), erythema, and joint swelling	Continue ICIs; NSAIDs (such as naproxen, 500 mg, twice a day, 4-6 weeks); if NSAIDs are not effective, consider using prednisone 10-20 mg/D for 2-4 weeks. If symptoms do not improve, upgrade to level 2 management treatment; consider steroid use in the affected joint based on the location of the joint and the number of affected joints.	Continue ICIs; acetaminophen and/or NSAIDs were used.
G2	Moderate pain with inflammatory symptoms, erythema, joint swelling; affect the ability to use tools of daily living (ADL)	Suspend ICIs; prednisone was used for 4-6 weeks, 0.5 mg/kg/d. If the symptoms did not improve, it was upgraded to level 3 management; if symptoms do not improve after 4 weeks, rheumatology consultation is recommended.	When the symptoms were improved and prednisone ≤ 10 mg/D, the use of ICIs could be resumed; larger doses of NSAIDs can be used as needed; consider intraarticular steroid injection; if the symptoms were not well-controlled, prednisone was used for 4-6 weeks, 10-20 mg/d; if the symptoms improve, gradually reduce within 4 to 6 weeks; if the symptoms do not improve, upgrade to level 3 management treatment; if the corticosteroid dose cannot be reduced to <10 mg/d after 3 months, consider DMARD.
G3	Severe pain with severe inflammatory pain, skin erythema or joint swelling; irreversible joint injury; disease; self-care ADL limitation	Suspend or permanently stop ICIs; prednisone for 4-6 weeks, 1 mg/kg/D; if the symptoms do not improve within 2 weeks, rheumatology consultation is recommended; according to the clinical phenotype of inflammatory arthritis, DMARD is considered to be used additionally. The available drugs include methotrexate, sulfasalazine, azathioprine, leflunomide, infliximab, and tuozhumab.	Suspend or permanently stop ICIs; if the symptom recovers to G1, continue the use of ICIs after consultation with the rheumatologist; prednisone was used for 4 weeks, 0.5-1 mg/kg/d; if symptoms do not improve or worsen after 4 weeks, consider using synthetic DMARD (methotrexate, leflunomide, and sulfasalazine) or biological DMARDs (TNF-*α* or IL-6 inhibitors).

**Table 3:** Management of immunotherapy-related polymyalgia rheumatica.

Level	Description	NCCN guideline
G1	Mild pain and/or stiffness, no limitation of ADL	Continue immunotherapy; prednisone, the initial dose of 5-20 mg/D ×6 weeks, then decreased in 4-6 weeks.
G2	Moderate pain and/or stiffness, affecting instrumental ADL	Stop immunotherapy; prednisone 10-20 mg/D, decreased in 8-12 weeks; if there is no improvement, please consult with the rheumatology department.
G3	Severe pain and/or stiffness, affecting self-care ADL	

**Table 4:** Management of immunotherapy-related myositis.

Level	Description	NCCN guideline	CSCO guideline
G1	Mild symptoms with or without pain	Consider stopping ICIs; consider PMR/GCA (see Table 3 for treatment principle); continuous monitoring of aldolase and creatine kinase; if indicated, treat pain (e.g., NSAIDs).	Continue ICIs; overall evaluation of patients' muscle strength; creatine kinase, aldolase, transaminase (AST, ALT), and lactate dehydrogenase (LDH) were monitored; if the level of creatine kinase increases and the muscle strength decreases, glucocorticoid can be given; after eliminating the related contraindications, acetaminophen or NSAIDs can be given for pain relief.
G2	Moderate symptoms with or without pain, affecting instrumental ADL	If the level rises, stop ICIs; muscle MRI and EMG were performed; prednisone 1-2 mg/kg/D; consider muscle biopsy, especially in severe or refractory cases; aldolase and creatine kinase were monitored continuously until symptoms disappeared or steroids were stopped.	ICI was suspended until the related symptoms were controlled, creatine kinase returned to the normal level, and prednisone dosage was less than 10 mg; NSAIDs can be given to relieve pain after removing related taboos; if creatine kinase ≥ 3 times of the upper limit of the normal value, prednisone (or equivalent dose of other drugs) was given for treatment.
G3	Severe symptoms with or without pain, affecting self-care ADL	If there are indications, treat the pain; please consult with the rheumatology department or neurology department; intravenous immunoglobulin (IVIG), 2G/kg, should be used for administration according to the instructions; if steroid is difficult to treat, plasma exchange may be considered and infliximab or mycophenolate mofetil may be given.	Suspend ICIs until G1; consider admission; please consult with the rheumatology department or neurology department; use 1 mg/kg/D methylprednisolone (or equivalent dose of other drugs); IVIG and plasma exchange were considered.
